# Multi-spatiotemporal projection enables single-shot ultrafast compressed imaging

**DOI:** 10.1038/s41377-026-02438-8

**Published:** 2026-08-18

**Authors:** Yingming Lai, Jinyang Liang

**Affiliations:** Laboratory of Applied Computational Imaging, Centre Énergie Matériaux Télécommunications, Institut National de la Recherche Scientifique, Université du Québec, Varennes, QC J3X 1P7 Canada

**Keywords:** Imaging and sensing, Ultrafast photonics

## Abstract

A multi-dimensional spatiotemporal projection-based ultrafast compressed imaging technique is proposed. By incorporating a multi-angle projection module via time-wavelength mapping and two-dimensional diffraction, omnidirectional spatial frequencies of the transient event are captured to eliminate spatial anisotropy and achieve ultrafast imaging with high spatiotemporal resolutions.

Ultrafast imaging technology plays a pivotal role in observing transient events across physics, biology, and materials science, as well as in unraveling their underlying mechanisms^1^. Although traditional multi-shot pump-probe techniques can achieve extremely high spatiotemporal resolutions, they rely on repetitive measurements and are applicable only to reproducible phenomena^2^. To capture non-repeatable or difficult-to-reproduce transient processes, single-shot ultrafast imaging technologies have emerged and rapidly evolved^3^. Among existing approaches, a notable technique is single-shot compressed streak imaging, which synergizes compressed sensing with streak imaging to record transient events in a single exposure with extraordinary imaging speed of up to hundreds of trillions of frames per second and a sequence depth of hundreds of frames^4–11^.

Despite its blooming development, existing single-shot compressed streak imaging systems are still limited by anisotropy in spatial resolution. Most systems use the single-dimensional spatiotemporal projection (SSP) architecture, which is implemented by temporally shearing the dynamic information of a transient phenomenon along one spatial axis. This approach leads to limited feature preservation in non-projection directions, which causes information loss and reconstruction artifacts of fine structures during the decoding process. Although numerous works have been developed to improve the imaging quality of SSP systems^12–18^, the limited omnidirectional spatial frequency sensing capability still presents a challenge to further enhancing spatial resolution.

To overcome these limitations, Prof. Feng Chen’s team at Xi’an Jiaotong University recently reported multi-dimensional spatiotemporal projection (MSP) ultrafast compressed imaging^19^. The core concept is the seamless integration of a multi-angle spatiotemporal data projection module into a conventional SSP system (Fig. 1a). In the experimental setup, an ultrashort chirped pulse first maps the spatiotemporal information of the dynamic scene to its spectrum. This modulated pulse is subsequently split into two paths. The first path enters an SSP module. A 2D diffraction grating replicates the scene into seven copies that are projected onto different regions of a binary encoding mask. The encoded scene is temporally streaked by a grating and spatiotemporally integrated by a CCD camera. This process forms seven images with the same projection angle but different encoding masks. The second path enters an MSP module. There, another 2D diffraction grating creates six replicas and shears each at a distinct angle (i.e., 30°, 90°, 150°, 210°, 270°, and 330°), ensuring the capture of omnidirectional spatial frequencies. Then, the recorded images from both the SSP and MSP modules are input into a two-step iterative shrinkage/thresholding algorithm based on total-variation regularization for image reconstruction.

**Fig. 1:**
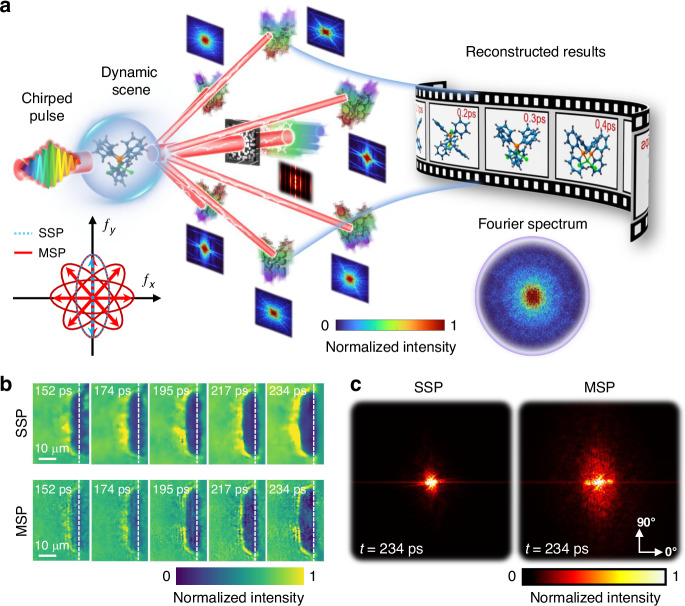
Single-shot ultrafast imaging by multiple-dimensional spatiotemporal projection (MSP). **a** Principle of MSP. **b** Femtosecond laser-induced ultrafast shock wave expansion captured by the single-shot single-dimensional spatiotemporal projection (SSP) and MSP ultrafast compressed imaging systems. **c** Fourier spectra of SSP and MSP at *t* = 234 ps. Adapted from ref. ^19^

Regarding system performance, static experiments demonstrate that the proposed method achieves a high resolution of 813 lp/mm in both vertical and horizontal directions. For dynamic imaging, with a sequence depth of up to 60 frames, the system maintains an omnidirectional spatial resolution exceeding 800 nm at an imaging frame rate of 0.3 trillion frames per second. Compared with traditional SSP systems, MSP improves the lateral resolution by 25% and the longitudinal resolution by 59–127%. Furthermore, MSP considerably mitigates image distortions and artifacts during dynamic reconstruction.

This technology has been successfully applied to high spatial resolution imaging of non-repeatable physical transients. As an example, in a side-view observation of shockwave expansion on a titanium alloy surface, MSP not only clearly reveals the shockwave front but also accurately captures a subtle convex structure at its center (Fig. 1b, c). This detail, unobservable with the SSP method, proves MSP’s much-enhanced imaging capability. The rich information in the captured video also enables new quantitative analysis of the shockwave’s axial distance over time. The resulting slope of 0.61 is consistent with the one-dimensional expansion behavior predicted by the Sedov-Taylor theory.

The ability to maximize recorded information during observation is central for achieving high-quality reconstruction in the compressed streak imaging paradigm. The introduction of MSP^20–23^ provides a thoughtful and complementary enhancement to this framework. In comparison with SSP, it enables new imaging capabilities, representing a significant advancement in single-shot ultrafast imaging. In the future, MSP ultrafast imaging is poised to make breakthrough advancements across hardware architecture, detection dimensions, and reconstruction algorithms. First, a chirped pulse with a broader spectral range (e.g., provided by supercontinuum light sources^24^) will be a direct path to improving system performance. A broader spectrum allows multiplexing more time-wavelength channels, thereby enhancing sequence depth and achieving superior spatiotemporal resolutions. Meanwhile, designing a single ultrathin metasurface device to simultaneously perform multi-order diffraction and precise focusing—while leveraging both the zeroth-order to achieve SSP and higher-order diffraction levels to perform MSP—could circumvent the space constraints of large-numerical-aperture lenses and bulky gratings, paving the way toward compact platforms^25^. Finally, the integration of advanced algorithms, such as PnP-ADMM and neural networks^26,27^, will not only drastically reduce reconstruction time for real-time applications but also enhance reconstruction fidelity under challenging low signal-to-noise ratio conditions. In summary, these frontier explorations in multi-physical dimensions and hardware-software synergy will complement the outstanding technical specifications of MSP ultrafast imaging, enabling it to play an irreplaceable role in observing a broader array of physical, chemical, and biological transient events.
